# Effects of distraction on taste-related neural processing: a cross-sectional fMRI study

**DOI:** 10.1093/ajcn/nqaa032

**Published:** 2020-03-16

**Authors:** Iris Duif, Joost Wegman, Monica M Mars, Cees de Graaf, Paul A M Smeets, Esther Aarts

**Affiliations:** 1 Radboud University, Donders Institute for Brain, Cognition, and Behavior, Nijmegen, Netherlands; 2 Division of Human Nutrition and Health, Wageningen University, Wageningen, Netherlands; 3 Image Sciences Institute and UMC Utrecht Brain Center, University Medical Center Utrecht, Utrecht, Netherlands

**Keywords:** distraction, attention, fMRI, taste, insula, orbitofrontal cortex, consumption

## Abstract

**Background:**

In the current obesogenic environment we often eat while electronic devices, such as smart phones, computers, or the television, distract us. Such “distracted eating” is associated with increased food intake and overweight. However, the underlying neurocognitive mechanisms of this phenomenon are unknown.

**Objective:**

Our aim was to elucidate these mechanisms by investigating whether distraction attenuates processing in the primary and secondary taste cortices, located in the insula and orbitofrontal cortex (OFC), respectively.

**Methods:**

Forty-one healthy, normal-weight participants received fixed amounts of higher- and lower-sweetness isocaloric chocolate milk while performing a high- or low-distracting detection task during fMRI in 2 test sessions. Subsequently, we measured ad libitum food intake.

**Results:**

As expected, a primary taste cortex region in the right insula responded more to the sweeter drink (*P* < 0.001, uncorrected). Distraction did not affect this insular sweetness response across the group, but did weaken sweetness-related connectivity of this region to a secondary taste region in the right OFC (*P*–family-wise error, cluster, small-volume corrected = 0.020). Moreover, individual differences in distraction-related attenuation of taste activation in the insula predicted increased subsequent ad libitum food intake after distraction (*r *= 0.36).

**Conclusions:**

These results reveal a mechanism explaining how distraction during consumption attenuates neural taste processing. Moreover, our study shows that such distraction-induced decreases in neural taste processing contribute to individual differences in the susceptibility for overeating. Thus, being mindful about the taste of food during consumption could perhaps be part of successful prevention and treatment of overweight and obesity, which should be further tested in these target groups. This study was preregistered at the Open Science Framework as https://bit.ly/31RtDHZ.

## Introduction

Worldwide, the prevalence of obesity has nearly tripled since 1975. In 2016, >1.9 billion adults were overweight, with 650 million being clinically obese (1). The problem of obesity has been partly attributed to the obesogenic food environment, which offers an enormous variety of palatable, energy-dense, easily consumed foods (2, 3). Furthermore, people's lifestyles have changed over the last decades, with increasing demands of multitasking due to their interaction with electronic devices [e.g., televisions, computers, and smart phones (4)]. As a consequence, people often eat while engaged in activities that prevent them from focusing on satiation signals such as sensory stimulation from the food products they are consuming or gastric signals [e.g., (2, 5)]. Such “mindless” or distracted eating has been causally related to increased immediate and later food intake and is associated with increases in BMI (2, 6–10).

However, the underlying neurocognitive mechanism of how distracted eating could increase food intake remains elusive. In rodents, others have found that distraction decreases and slows down processing of taste information in the taste cortex (11). In humans, it has been suggested that distraction attenuates taste perception due to limited attentional capacity, which then leads to overconsumption (12). However, this putative mechanism of how distraction during taste processing relates to overeating has never been tested. One study investigated effects of cognitive load on food reward–related processing (13); however, this study did not assess brain responses to actual consumption of food. An increased understanding of the neurocognitive mechanism in humans could not only reveal the different factors influencing distraction-related overeating but may also shed light on individual differences in the susceptibility for overeating.

We hypothesized that distraction attenuates processing in the primary and secondary taste cortices, located in the insula and orbitofrontal cortex (OFC), respectively [see, e.g., (8)]. The primary taste cortex has been associated with identification, pleasantness, and intensity of tastes (8, 14–16). The OFC receives direct input from the primary taste regions in the insula and has been related to reward-related taste processing, such as hedonic evaluation (15, 17, 18). Satiety modulates processing in both the primary and secondary taste cortices; both regions show greater taste activation in a state of hunger (19–21). Thus, distraction during food consumption—e.g., due to multitasking—might affect processing of primary and higher order taste regions (primary outcome measure) and their connectivity (secondary outcome measure), resulting in attenuated processing of satiety signals (secondary outcome measure: fullness) and increased food intake (secondary outcome measure).

## Methods

### Participants

Forty-six right-handed healthy adults, who were recruited from Nijmegen and the surroundings through advertisement, participated in the study. They gave written informed consent and were reimbursed for participation according to institutional guidelines of the local ethics committee (Commissie Mensgebonden Onderzoek region Arnhem-Nijmegen, Netherlands, 2015-1928). A flowchart of the study is shown in **Supplemental Figure 1**. As a result of dropout [i.e., not completing the second test session (*n* = 1), technical problems (*n* = 4)], the final sample size of the study was 41 [age range: 18–35 y; mean ± SD age: 22.5 ± 3.5 y; 31 women; mean ± SD BMI (kg/m^2^): 21.9 ± 1.89; mean ± SD waist-to-hip ratio (WHR): 0.80 ± 0.05]. Before the study, a sample-size calculation (see preregistration) was performed and approved by the local ethics committee.

### Procedure

**FIGURE 1:**
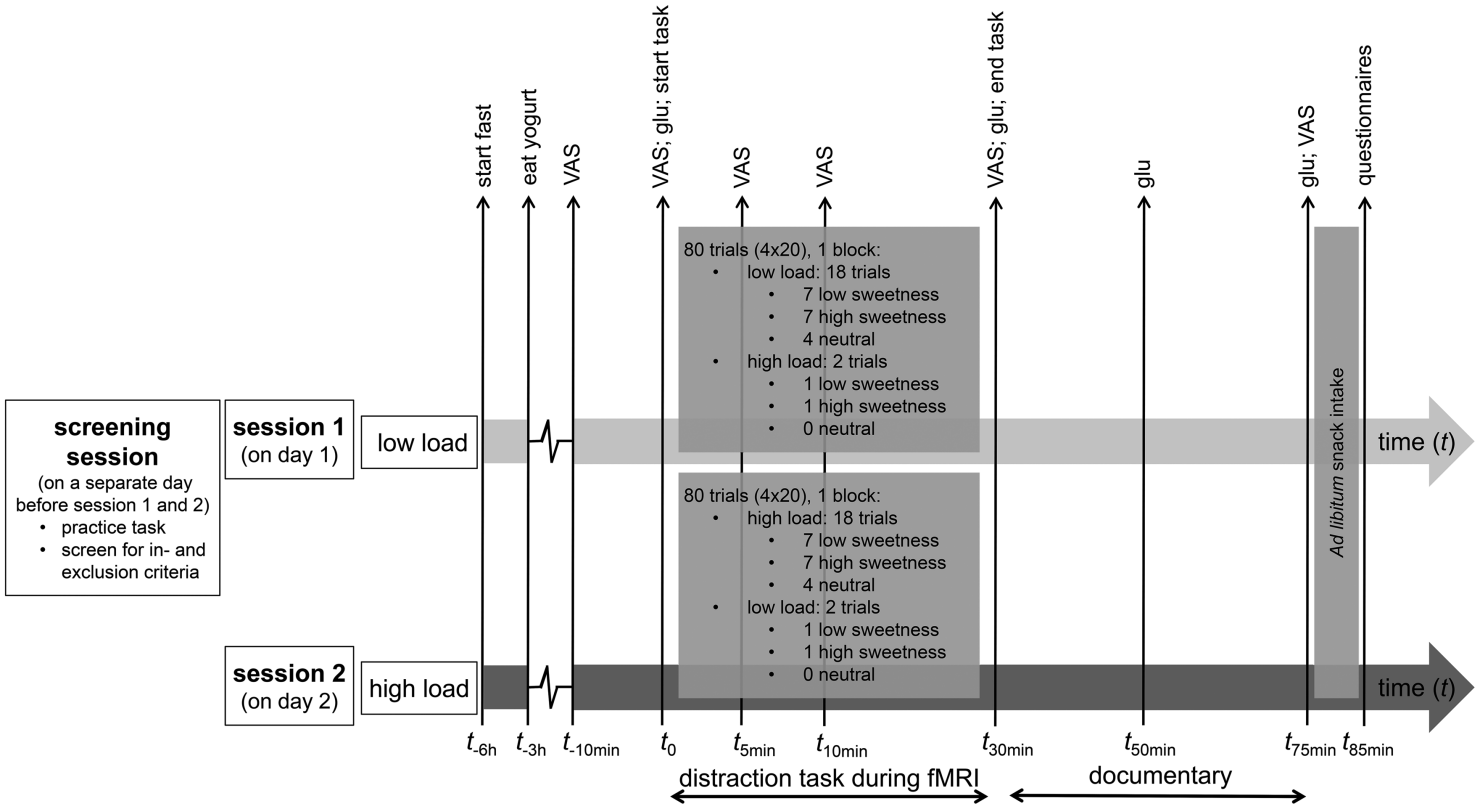
Timeline of the experimental sessions. After a screening session, participants came to the laboratory twice, for 2 experimental sessions. Except for the difference in attentional load (high or low) during the distraction task, the 2 sessions were identical. Session order was counterbalanced across participants. Between *t*_0_ and *t*_30_, participants performed the high- or low-distraction task during fMRI scanning. In each session, participants performed 80 trials (4 blocks of 20 trials). To manipulate distraction, 90% of trials were of low load (high-frequency trials) and 10% of high load (low-frequency trials) in the low-load session, and vice versa for the high-load session. Each block had 8 trials of low sweetness, 8 of high sweetness, and 4 of neutral taste. After the task, participants were removed from the MR scanner and watched a documentary in the behavioral laboratory until *t*_75_. Subsequently, participants consumed a chocolate snack ad libitum. Glucose (glu) measurements and VAS hunger, fullness, thirst, ideal sweetness, liking, sweet and savory desire, nausea, and anxiety were rated at several time points. See the Methods section for further details. MR, magnetic resonance; VAS, visual analog scale.

During a screening session (**Figure 1**), anthropometric measurements [BMI (weight in kilograms/height in centimeters squared) and WHR (waist in centimeters/hip in centimeters)] were obtained. Participants practiced the task (see below) to avoid between-session effects, and filled out questionnaires to screen for inclusion and exclusion criteria (for a detailed description see **Supplemental Methods**, Inclusion and exclusion criteria).

Upon inclusion, participants were invited to the laboratory at the Donders Centre for Cognitive Neuroimaging (Nijmegen) for 2 experimental test sessions: a low- and a high-distraction session (for an overview of the sessions, see Figure 1). Session order was randomly assigned: half of the participants had the low-distraction session first and the high-distraction session second, the other half of participants had the inverse order. Prior to each test session, participants were instructed to abstain from eating solid foods and from drinking sugared or sweetened drinks (but not water) 6 h prior to the experiment and to refrain from alcohol use (24 h) and neuroleptic or psychotropic drug use (7 d). To standardize participants’ hunger, they were instructed to ingest a standardized load 3 h before each session (yogurt drink, strawberry flavor: 200 g; 850 kJ, 6.0 g protein, 30.0 g carbohydrates, 6.0 g fat; Breaker; Melkunie).

**FIGURE 2:**
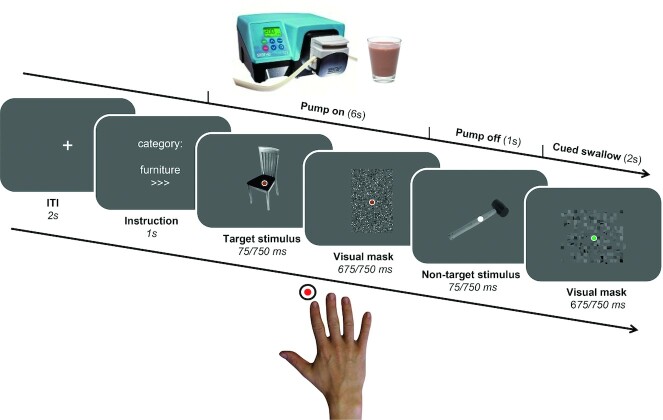
Trial structure of the categorical visual detection task. Each trial started with an instruction screen, indicating the target category (furniture, tool, or toys) and attentional load (low “>” or high “>>>”) of the trial. Then, pictures were presented followed by a visual mask, and subjects were instructed to push a button as fast as possible upon detection of pictures belonging to the instructed category. During each trial, participants were administered a fixed amount of lower- or higher-sweetened chocolate milk, or a tasteless neutral solution through a gustometer. Markers were placed on the participant's neck to enable detection of participants’ swallow movements. Onsets and offsets of the swallow movements were used to determine trial durations in the first level (single-subject) fMRI models. ITI, inter-trial interval.

At the start of the first test session, anthropometric measurements were taken. Subsequently, participants underwent an fMRI scan for 30 min in which they performed a categorical visual detection task (Figure 1 and **Figure 2**). During the task, participants received higher-sweetness (120 g) or lower-sweetness (120 g) chocolate milk, or a tasteless solution (60 g), through small tubes. After scanning, participants watched a documentary (BBC Life, “Primates or Plants”; order was randomized). Subsequently, participants were seated in front of a bowl with colored button-shaped chocolates (M&Ms; Mars Wrigley) for 10 min, and were asked to eat until comfortably full. In session 1, the participants completed the Behavioural Inhibition System/Behaviour Approach System (22), the Baratt Impulsiveness Scale-11 (23), and Kirby (delayed reward discounting) (24) questionnaires. In session 2, they completed the following questionnaires: the Binge Eating Scale (25), Food Frequency Questionnaire–Dutch Healthy Diet (26), Dutch Eating Behaviour Questionnaire (27), and the Power of Food Scale (28), which are used to describe the study population (see **Supplemental Table 1** for mean scores and SDs).

During the test session, glucose measurements were taken through finger pricks and analyzed with use of a glucose meter (Stat Strip Xpress®; Nova Biomedical). This was done at 4 time points (*t*): *t*_0min_ (baseline, before first chocolate milk exposure), *t*_30min_ (directly following last exposure to chocolate milk), *t*_50min_, and *t*_75min_ (before consumption of the chocolate snack).

At several time points during the experiment, participants rated how hungry, full, and thirsty they were on a paper (*t*_-10min_and *t*_75min_) or digital (*t*_0min,_*t*_5min_, *t*_10min_, and *t*_30min_) version of a visual analog scale (VAS; 100-mm line), with anchors ranging from 0 (“not at all”) to 10 (“very”). Before and after the task (*t*_0min_ and *t*_30min_), participants also digitally rated how nauseous and anxious they felt, and their desire for something savory and something sweet.

At least 1 wk after their first test session (mean difference ± SD: 11.93 ± 7.92 d), participants revisited the laboratory for their second test session. The time of day at which participants had their experimental sessions varied between subjects (minimum: 10:45 h; maximum: 19:10 h). Within-subject, we aimed to plan a participant's second session at a similar time of day as his/her first session (mean ± SD time difference: 1.00 ± 1.52 h).

### Gustatory stimulation

Gustatory stimuli were determined in a pilot study (see Supplemental Methods: Gustatory pilot study). The higher- and lower-sweetness chocolate milk were solutions of cocoa powder (Blooker; Bickery Food Group; 2 g), dextrine-maltose (Fantomalt; Nutricia; 9 g) in whole milk (3.5% fat/100 g), and liquid noncaloric sweetener (Natrena; Jacobs Douwe Egberts B.V.; lower sweetness: 0.0867 g; higher sweetness: 1.5457 g/100 g whole milk). The neutral solution was determined based on work by Veldhuizen et al. (29), and contained 1.25 mol/m^3^ sodium bicarbonate and 12.5 mol/m^3^ potassium chloride in water.

Before and after the task that participants performed in the magnetic resonance (MR) scanner, they received 2 rounds of 3.75 g of the neutral drink and of the lower- and higher-sweetness drink for tasting through tubes innervated by pumps (gustometer; Watson-Marlow). For all drinks, they rated how much they liked the drink on VASs. For the 2 chocolate milk drinks, they also rated to what extent the sweetness of the chocolate milk matched their ideal sweetness on an ideal point scale.

### Categorical visual detection task

Participants performed a categorical visual detection task during 3T fMRI scanning (Figure 2). The task was programmed with Presentation software (version 16; Neurobiobehavioral Systems, Inc.). Each trial (total duration: 12 s) started with a fixation cross (duration, 2 s), followed by an instruction screen (1 s), indicating the category of pictures to which the participant needed to respond (furniture, tools, or toys) and the speed of the trial (“>” for a slow trial, “>>>” for a fast trial). For example, if the instruction screen stated “category: furniture, >>>,” this meant that participants needed to respond to stimuli in the category of furniture, and the pictures would be presented at high speed. In order to keep visual stimulation equal for both trial types, a visual mask always followed a picture. The visual masks were scrambled versions of the stimulus pictures, to keep luminance equal. For the low-speed trials, both pictures and visual masks were presented for 750 ms. For the high-speed trials, pictures were presented for 75 ms and the visual mask for 675 ms. Consequently, there were twice as many pictures and visual masks in the high-speed trials relative to the low-speed trials (12 vs. 6), thus a higher attentional load. Whenever a target stimulus was presented (i.e., a picture belonging to the instructed category), participants had to push a button upon detection with their right index finger. Participants made responses using an MRI-compatible button box. Participants received no feedback on whether they responded correctly.

During the trials, participants received a fixed amount (3.75 g) of chocolate milk of higher or lower sweetness or a fixed amount of the neutral solution through a gustometer. Drink administration started together with presentation of the first picture and lasted for 6 s (i.e., a flow rate of 0.625 g/s). The drinks were administered at room temperature. A dot changing in color in the center of the screen informed participants of the start (brown color) and finish (white color; 1 s) of administration. At the end of each trial, the dot turned green (2 s), cueing participants to swallow the milk or tasteless solution. As swallowing can also be an uncontrollable, reflexive movement, participants’ swallowing was filmed. A marker was placed on the Adam's apple, as this area shows the most swallow-related movement. Frame-by-frame video analysis of the marker's movement was later performed to pinpoint the exact moments in time when participants swallowed during the experiment (see Supplemental Methods: Video analysis of swallow movements*)*.

Participants performed 4 blocks of 20 trials (a total of 80 trials; see Figure 1). For the low-distraction session, 90% of the trials were low-speed trials (high-frequency trials: pictures presented at a slow pace), and 10% of the trials were high-speed trials (low-frequency trials: pictures presented at a fast pace). Thus, in the low-distraction session, each block contained 18 low-speed trials and 2 high-speed trials. For the high-distraction session, this division was the same, although in the opposite direction [90% high-difficulty trials (high-frequency trials), 10% low-difficulty trials (low-frequency trials)]. The low-frequency trials were added to ensure participants would keep anticipating the occurrence of both high- and low-speed trials in both sessions. Each block of 20 trials had 4 neutral taste trials. Trials 1, 7, 14, and 20 were always neutral. Of the remaining trials, 7 trials (50%) were of higher sweetness and 7 (50%) of lower sweetness. Category (whether participants had to respond to pictures in the category tools, toys, or furniture) and drink sweetness presentation were pseudo-randomized (i.e., the same category and sweetness were never presented >3 times in a row). Moreover, maximally 2 target stimuli (i.e., pictures belonging to the instructed category) were presented after another.

### MRI data acquisition and analysis

To measure BOLD contrast, whole-brain functional images were acquired on a Siemens 3T Skyra MRI scanner (Siemens Medical Systems) using a 32-channel coil. During the task, 3D echo planar imaging scans using a T2*-weighted gradient echo multi-echo sequence (30) were acquired [voxel size: 3.5 × 3.5 × 3 mm isotropic, TR (repetition time) = 2070 ms, TE (echo time) = 9 ms; 19.25 ms; 29.5 ms; 39.75 ms; field of view (FoV) = 224 mm]. The slab positioning and rotation (average angle of 14° to the anterior commissure axis) optimally covered both prefrontal and deep brain regions. Before the acquisition of functional images, a high-resolution anatomical scan was acquired [T1-weighted magnetization-prepared rapid gradient-echo (MPRAGE) sequence: voxel size 1 × 1 × 1 mm, TR 2300 ms, TE 3.03 ms, 192 sagittal slices, flip angle 8°, FoV 256 mm].

fMRI data were preprocessed using SPM8 (www.fil.ion.ucl.ac.uk/spm) and FSL version 5.0.11 (http://www.fmrib.ox.ac.uk/fsl/). The volumes for each echo time were realigned to correct for motion artefacts (estimation of the realignment parameters is done for the first echo and then copied to the other echoes). The 4 echo images were combined into a single MR volume based on 30 volumes acquired before the actual experiment started using an optimized echo-weighting method (30). Combined functional images were slice-time corrected by realigning the time series for each voxel temporally to acquisition of the middle slice and spatially smoothed using an isotropic 8-mm full-width at half-maximum Gaussian kernel. Next, Independent Component Analysis based Automated Removal of Motion Artifacts (ICA-AROMA) (31) was used to reduce motion-induced signal variations in the fMRI data. Subject-specific structural and functional data were then coregistered to a standard structural or functional stereotactic space [Montreal Neurological Institute (MNI) template], respectively. After segmentation of the structural images using a unified segmentation approach, structural images were spatially coregistered to the mean of the functional images. The resulting transformation matrix of the segmentation was then used to normalize the anatomical and functional images into MNI space. The functional images were resampled at voxel size 2  × 2 × 2 mm.

### Statistical analysis

To test for pre-experimental differences in liking of the higher- compared with the lower-sweetness chocolate milk, we performed a repeated-measures ANOVA (IBM SPSS Statistics 23) with within-subject factor drink sweetness (lower sweetness, higher sweetness) on the mean baseline ratings.

We used the sensitivity index *d′* to calculate participants’ task performance (see Supplemental Methods “*d prime*” for how this score was calculated). Mean *d′* scores on the detection task were analyzed using repeated-measures ANOVA with attentional load (low, high) and drink type (lower-, higher-sweetness milk, neutral drink) as within-subject factors. Low-frequency conditions (i.e., 10% low-speed trials on the high-distraction session and vice versa) were excluded from this analysis, as the low number of trials in these conditions would likely bias the results.

Statistical analysis of fMRI data was performed using a general linear model approach in SPM8 (www.fil.ion.ucl.ac.uk/spm). The images of both experimental runs were combined into 1 model including the low- and high-distraction test sessions. At the individual (first) level, subject-specific data were analyzed using a fixed-effects model, which included 10 regressors of interest. For each experimental run, the first 4 regressors reflected the trials of low attentional load, lower-sweetness drink; low attentional load, higher-sweetness drink; high attentional load, lower-sweetness drink; and high attentional load, higher-sweetness drink. The fifth regressor reflected trials in which the neutral solution was given, which was always of high-frequency load, so of low attentional load in the low-distraction session and of high attentional load in the high-distraction session to rinse in-between chocolate milk trials. Durations reflected the moment the gustometer started drink administration until the first swallow of the participants, or in case these video data were not available, the moment the swallow was cued in the task (dot turning green, mean ± SD duration: 11.22 ± 0.80 s). All regressors were convolved with the canonical hemodynamic response function. Parametric modulators reflecting the number of button presses per trial were added to the model for each regressor of interest, to correct for signal change induced by the difference in number of targets between the low and high attentional load condition. High-pass filtering (128 s) was applied to the time series of the functional images to remove low-frequency drifts, and correction for serial correlations was done using a first-order autoregressive (AR-1) model. Signal variation in white matter and cerebrospinal fluid regions was also included.

At the group (second) level, we assessed the effect of load (high > low attentional load) in 2 ways. First, we contrasted high- with low-load trials by including the high-frequency trials of each test session only, meaning a contrast (comparison) between sessions (high-distraction session: high-load regressors across drink types > low-distraction session: low-load regressors across drink types). Second, we also added the low-frequency regressors to assess this contrast (high- + low-distraction session: high-load regressors across drink types > low-load regressors across drink types).

Taste-related brain areas (sensitive to sweetness) were localized with the contrast higher > lower sweetness (*P *< 0.001, uncorrected), over both test sessions. For this contrast, within-test session data were available; therefore, we did not make a second contrast including low-frequency regressors. We used the Automated Anatomical Labeling (AAL) atlas (32) to determine whether the areas activated in this functional contrast overlapped with the anatomical insula (bilateral insula) and OFC (bilateral superior, medial, mid-, and inferior orbitofrontal regions of this atlas).

To investigate the effect of attentional load on processing in primary and secondary taste-related areas, we assessed the interaction effect of load (high > low load) × sweetness (higher > lower) using the test sessions in which the load conditions were the high-frequency load condition. The activated taste-related regions (determined by the contrast higher > lower sweetness) were used as regions-of-interest (ROIs) for the load × sweetness interaction contrast. Mean parameter estimates (i.e., β weights) were extracted from all voxels in both ROIs separately using MarsBar (33). The regionally averaged parameter estimates were analyzed using ANOVA with the same factors as in the whole-brain analyses. As 2 ROIs were tested, effects were considered significant when reaching a threshold of *P *< 0.025 (Bonferroni-corrected for multiple comparisons). In exploratory analysis, we also assessed the interaction effect of load and sweetness at the whole-brain level [on a *P *< 0.001 uncorrected and a whole-brain *P* family-wise error (FWE) cluster < 0.05 corrected threshold].

As a secondary analysis, we performed a generalized psychophysiological interaction (gPPI) (34) analysis to investigate distraction-related differences in functional connectivity for taste-related processing. As a seed, we used a taste-related region from our localizer approach described above. To estimate the neural activity producing the physiological effect in the seed region for each subject, the BOLD signal was extracted from this region and deconvolved (35). This was included in the model as the physiological regressor. The durations for each of the relevant task conditions—*1*) low-load, lower-sweetness drink; *2*) low-load, higher-sweetness drink; *3*) high-load, lower-sweetness drink; and *4*) high-load, higher-sweetness drink—were included as psychological regressors. The psychophysiological interaction was entered by multiplying the estimated neural activity in the seed region by the duration times for each of the task conditions, separately convolved with the hemodynamic response function, resulting in 9 regressors of interest on the first level (i.e., 1 physiological, 4 psychological, and 4 interaction regressors). For each subject, we created a psychophysiological interaction (PPI) contrast for the interaction effect of distraction (high > low attentional load) and sweetness (higher > lower sweetness). On the second level, this PPI contrast was analyzed separately using a 1-sample *t* test. Statistical inference (*P*_FWE _< 0.05) was performed at the cluster level, correcting for multiple comparisons over the a priori–defined small-search volume: bilateral insula and OFC [AAL atlas (32)].

The results of all random-effects fMRI analyses had a threshold of *P* < 0.001 (uncorrected) and statistical inference was performed at the cluster level, FWE-corrected (*P*_FWE _< 0.05) for multiple comparisons over the search volume (the whole-brain or a priori–defined small-search volumes).

The effect of attentional load on subsequent ad libitum food intake was tested with a paired-samples *t* test to compare the total amount of chocolate snacks consumed (grams) between the high- and low-distraction test sessions.

Blood-glucose concentrations were analyzed using the linear mixed model [lmer4, version 1.1-14 (36); package in R, version 3.5.1; https://www.r-project.org], because we expected variation across participants in the glucose response over time, and traditional repeated-measures ANOVA cannot account for such variation. Participants’ glucose concentrations were analyzed with time (*t* = 0 min, *t* = 30 min, *t* = 50 min, and *t* = 75 min) and load (low, high) as fixed factors. We used random intercepts for participants, as we expected fasted glucose concentrations to vary between participants. Moreover, we used random slopes for the time predictor to account for the expected variation across participants in glucose response over time.

To test whether mean appetite (hunger, fullness) and thirst ratings varied as a function of load, we used repeated-measures ANOVA with within-subject factors load and time (digital: *t*_0_, *t*_5_, *t*_10_, *t*_30_, paper: *t_-_*_5_, *t*_75_).

In an exploratory analysis, we investigated whether ad libitum food intake, blood glucose concentrations, and appetite and thirst measures (filled out digitally or on paper) covaried significantly with the effect of load on processing in the taste-related areas differentially activated in the higher > lower sweetness contrast. For ad libitum food intake, a repeated-measures ANCOVA (IBM SPSS Statistics 23) was executed with load (high, low) and sweetness (higher, lower) as within-subject factors and the difference in food intake between the low- and high-distraction sessions as a covariate. For the blood glucose measurements, we performed an ANCOVA with glucose as a dependent variable and load (high, low) and time (*t*_0_, *t*_30_, *t*_50_, *t*_75_) as within-subject factors. The difference in brain responses (higher–lower sweetness) between the low- and high-distraction session was used a covariate. For the hunger, fullness, and thirst digital and paper ratings, the difference over time (digital: *t*_30_–*t*_0_, paper: *t*_75_–*t-*_5_) between the low- and high-distraction session was used for the covariates. Finally, we performed some additional analyses on the liking, ideal sweetness, and sweet and savory desire ratings (see Supplemental Methods and **Supplemental Results**: Liking and ideal sweetness ratings and Desire for something sweet or savoury; **Supplemental Table 2**). The study was executed in accordance with good practice standards as described in Smeets et al. (37).

## Results

### Initial liking of gustatory stimuli

At baseline, participants liked the higher-sweetness drink significantly more than the lower-sweetness drink [main effect of drink sweetness: higher-sweetness drink, mean (SE): 6.1 (0.3); lower-sweetness drink, mean (SE):  5.5 (0.3); variance ratio: *F*(1,34) = 4.53; *P*  = 0.041; Supplemental Table 2). Therefore, all following fMRI results were corrected for this pre-experimental difference by adding it as a covariate to the analyses. None of the effects below (activation or connectivity) covaried with initial liking (all *P* > 0.1). The lower-sweetness drink was perceived equally far from participants’ ideal sweetness as the higher-sweetness drink (Supplemental Results: Liking and ideal sweetness ratings; Supplemental Table 2).

### Performance under distraction

Resulting from our distraction manipulation, participants detected fewer targets when they were rapidly presented [i.e., during the high-frequency trials (90% high-load trials) in the high-distraction session (*d′* ± SEM: 2.43 ± 0.10)] than when they were slowly presented [i.e., during the high-frequency trials (90% low-load trials) in the low-distraction session (*d′* ± SEM: 4.00 ± 0.10; *F*(1,40) = 264.11; *P* < 0.001]. As expected, the difference in sweetness did not affect performance [drink sweetness (lower, higher): *F*(1,40) <1; *P* = 0.748; drink sweetness × load: *F*(1,40) <1; *P* = 0.907].

### fMRI results

Before testing whether the effect of distraction, operationalized as attentional load, affected neural taste processing, we first determined whether our load and sweetness manipulations activated the expected brain regions [i.e., fronto-parietal attention network, e.g., (38), and insula/OFC, respectively].

On our whole-brain–corrected threshold [*P*_FWE_ (cluster-level) < 0.05], we only found effects of attentional load in a visual and a temporal region when taking solely the high-frequency trials (90% high-load trials vs. the 90% low-load trials) into account [i.e., when comparing between test sessions (high- > low-distraction session)]. However, when contrasting between the high- and low-distraction sessions, while also taking the low-frequency trials into account (high- + low-distraction session: high-load trials > low-load trials, across drink types), we found more extensive responses in areas typically activated in tasks varying in attentional load, including visual and fronto-parietal regions (**Table 1**).

**TABLE 1:** Summary of brain regions exhibiting effects of distraction (attentional load), sweetness, interactions between distraction and sweetness, and the result of the connectivity analysis (gPPI)^1^

Label	Side (left/right)	Part of cortex (F, P, T, O, I, C)	MNI coordinates x, y, z, mm			Size, no. of voxels	*P*_FWE_ (cluster-level)	*t* value (peak)
Effect of distraction (high > low attentional load; low-frequency regressors included)^2^								
Calcarine	L	O	−8	−100	2	2532	<0.001	8.57
Calcarine	R	O	14	−92	0	—	—	8.20
Cerebellum	R	C	8	−78	−16	—	—	8.06
Superior motor area	L	F	−4	10	52	2183	<0.001	7.89
Frontal medial lobe	L	F	−30	−4	52	—	—	7.13
Mid cingulum	R	F	6	18	46	—	—	6.72
Parietal superior lobe	R	P	24	−56	50	376	0.003	7.63
Precentral	R	F	42	4	30	1177	<0.001	7.28
Frontal medial lobe	R	P	30	4	60	—	—	6.21
Precentral	R	F	42	0	46	—	—	5.65
Temporal medial lobe	R	T	44	−66	8	788	<0.001	7.21
Medial occipital lobe	R	O	32	−70	22	—	—	5.37
Cerebellum	L	C	−38	−56	−34	444	0.001	7.14
Insula	L	I	−32	22	4	1106	<0.001	6.91
Inferior frontal gyrus, orbital	R	F	48	26	−6	—	—	6.33
Inferior frontal gyrus, triangular	R	F	58	22	8	—	—	3.93
Medial occipital lobe	L	O	−42	−72	6	625	<0.001	6.82
Parietal superior lobe	L	P	−20	−62	52	228	0.028	5.93
Insula	L	I	−30	24	0	466	0.001	5.66
Insula	L	I	−34	20	10	—	—	4.80
Insula	L	I	−32	30	10	—	—	4.74
Effect of distraction (high > low attentional load; low-frequency regressors excluded)^2^								
Temporal medial lobe	R	T	54	−62	10	210	0.043	4.37
Calcarine	L	O	2	−88	−6	350	0.005	4.37
Effect of distraction (low > high attentional load; low-frequency regressors included)^2^								
Inferior occipital lobe	R	O	38	−88	−10	6611	<0.001	8.70
Cuneus	R	O	10	−70	22	—	—	8.66
Calcarine	R	O	20	−64	10	—	—	8.18
Postcentral	R	P	60	−8	26	3074	<0.001	7.78
Rolandic operculum	R	F, P	48	−6	20	—	—	7.73
Postcentral	R	P	56	−12	34	—	—	7.58
Fusiform area	L	T, O	−36	−82	−12	693	<0.001	7.36
Medial occipital lobe	L	O	−36	−92	−6	—	—	6.28
Postcentral	L	P	−60	−16	38	3082	<0.001	7.31
Parietal inferior lobe	L	P	−52	−34	44	—	—	7.21
Postcentral	L	P	−54	−14	32	—	—	7.04
Sweetness localizer, higher > lower sweetness, masked with AAL insula + OFC^3^								
Insula	R	I	38	−4	10	12	0.373	4.69
Insula	L	I	−34	−6	14	2	0.565	3.37
gPPI—interaction effect [distraction (low > high) > sweetness (higher > lower)]^4^								
Inferior frontal gyrus, orbital	R	F	32	28	−18	116	0.020	5.27

1 For the main effects of distraction, results are shown for the comparisons including and excluding the low-frequency regressors (see Methods). AAL, Automated Anatomical Labeling; C, cerebellar cortex; F, frontal cortex; FWE, family-wise error; gPPI, generalized psychophysiological interaction; I, insular cortex; L, left; MNI, Montreal Neurological Institute; O, occipital (visual) cortex; OFC, orbitofrontal cortex; P, parietal cortex; R, right; T, temporal cortex.

2 *P* < 0.05, whole-brain FWE corrected.

3 *P* < 0.001, uncorrected. Areas showing overlap with a priori*–*defined AAL atlas regions.

4 *P* < 0.05, small volume, FWE corrected.

**FIGURE 3:**
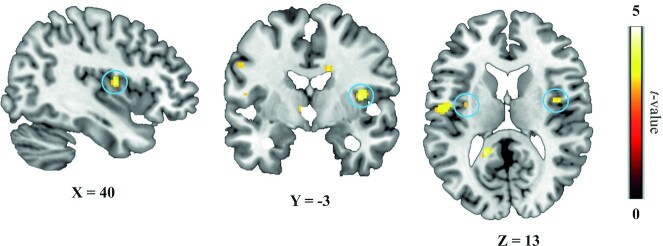
BOLD responses to the effect of sweetness. The left (2 voxels) and right (12 voxels) middle insula show larger responses to the higher- vs. lower-sweetness drink comparison (circled in blue; *n* = 41). Statistical parametric maps had a threshold of *P* < 0.001 uncorrected for visualization purposes. All statistical parametric maps were overlaid onto a T1-weighted canonical image. Slice coordinates are defined in MNI152 space and images are shown in neurological convention (left = left). MNI, Montreal Neurological Institute.

Next, we localized brain regions responding to the difference in sweetness of the chocolate milk (higher > lower sweetness; *P *< 0.001, uncorrected). As expected, within our a priori–defined search volume (i.e., the anatomically defined bilateral insula + bilateral OFC from the AAL atlas), clusters in the left and right insula responded to this difference (Table 1 and **Figure 3**). As the left cluster comprised only 2 voxels, further analyses focused on the right functional insula cluster for the effects of distraction. We did not find differential sweetness responses in the OFC.

**FIGURE 4:**
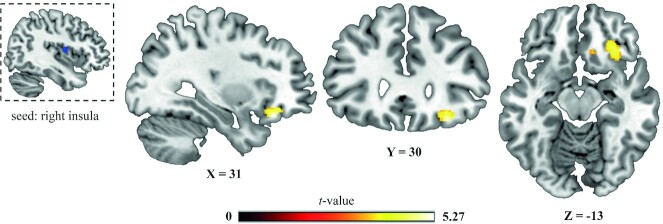
Results of the gPPI analysis with the right insula seed region in the top left (in blue, extracted from the higher- > lower-sweetness comparison). Shown is the right orbitofrontal area exhibiting significantly (*P* = 0.020 after SVC) higher sweetness-related functional connectivity with the seed region under low, relative to high, distraction. *n* = 41. gPPI, generalized psychophysiological interaction.

Finally, we tested the effect of distraction (attentional load) on activation of the right insula cluster that responded to the sweetness manipulation. We found no evidence for this effect, as the averaged extracted condition parameter estimates across the right insula cluster did not show a load × sweetness interaction [*F*(1,40) <1, *P* = 0.711]. We obtained similar results when we explored the interaction effect of load and sweetness at the whole-brain level, where we did not find any whole-brain–corrected results (*P*_FWE _< 0.05; see **Supplemental Figure 2** for the results at *P *< 0.001, uncorrected). However, when we used this right insula cluster that showed greater responses for high > low sweetness (at *P *< 0.001, uncorrected) as a seed in the functional connectivity (i.e., gPPI) analysis, it showed decreased connectivity with a region in the right OFC under high compared with low distraction during processing of sweet taste (higher > lower sweetness; Table 1 and **Figure 4**). This right OFC region (MNI-coordinates x, y, z (mm): 32, 28, −18; AAL: right inferior frontal gyrus, pars orbitalis) was significant within our a priori–defined search volume of the insula plus OFC [*P*_FWE_ (cluster, after small volume correction) = 0.020, *t* = 5.27, *k* = 116]. This shows that distraction weakens functional connectivity between the right insula and right OFC during taste processing.

### Effect of distraction on appetite and thirst ratings

Hunger, fullness, and thirst ratings that were filled out digitally during the task (*t*_0_, *t*_5_, *t*_10_, *t*_30_) and on paper before and after the test session (*t*_-5_, *t*_75_) were analyzed separately (**Table 2**). All ratings showed main effects of time, except for hunger, which was not significant in the paper ratings. These effects indicate significant increases in fullness and significant decreases in thirst and hunger over the time course of the task (digital) or test session (paper), as anticipated. No effects of distraction (i.e., attentional load) on any of the ratings were found. In addition, we found no significant pre-experimental differences between the low- and high-distraction session for all self-reported measures.

**TABLE 2:** Appetite (hunger, fullness) and thirst ratings, filled out digitally and on paper, averaged over distraction (high, low attentional load)^1^

	*t*_(-5)_	*t*_(0)_	*t*_(5)_	*t*_(10)_	*t*_(30)_	*t*_(75)_	*P*
Digital appetite and thirst ratings							
Hunger	—	6.8 (0.2)	6.4 (0.2)	5.9 (0.3)	5.9 (0.3)	—	0.005
Fullness	—	2.2 (0.2)	2.9 (0.3)	3.7 (0.4)	4.4 (0.4)	—	<0.001
Thirst	—	6.0 (0.3)	4.2 (0.4)	3.6 (0.4)	3.8 (0.4)	—	0.001
Appetite and thirst ratings on paper							
Hunger	6.3 (0.2)	—	—	—	—	6.4 (0.2)	NS
Fullness	2.3 (0.2)	—	—	—	—	3.6 (0.3)	<0.001
Thirst	5.3 (0.3)	—	—	—	—	4.3 (0.3)	0.004

1 Values are means (SEs) per time point and time statistics. The reported *P* values were obtained using repeated-measures ANOVA with the within-subject factor time (digital: *t*_0_, *t*_5_, *t*_10_, *t*_30_; paper: *t*_-5_, *t*_75_). *n* = 41.

### Blood glucose concentrations

For 4 participants, one of the glucose measurements failed. Therefore, these measurements were excluded from the analyses. Analysis of the blood glucose concentrations revealed a main effect of time [*t*(1, 88.85) = 11.28, *P* < 0.001; **Supplemental Figure 3**], meaning that participants’ blood glucose concentrations increased significantly over the 4 time points during and after chocolate milk consumption, as expected [mean (SE): *t*_0_ = 4.43 (0.46) mmol/L, *t*_t30_ = 4.84 (0.74) mmol/L, *t*_50_ = 7.14 (1.23) mmol/L, *t*_75_ = 7.17 (1.29) mmol/L]. Furthermore, distraction (attentional load) did not affect these glucose increases over time [*t*(1, 260.19) = 1.81, *P* = 0.072]. However, an exploratory analysis did reveal a significant distraction-related decreased rise in glucose concentrations at *t* = 75 relative to baseline [mean (SE): low-distraction session: *t*_75_–*t*_0_ = 2.94 (1.48) mmol/L; high-distraction session: *t*_75_–*t*_0_ = 2.50 (1.32) mmol/L; *t*(1, 113.96) = 2.09; *P* = 0.039]. Nevertheless, further post hoc analyses (see Supplemental Results: Blood glucose concentrations) showed that these small effects were not statistically reliable. Given the low physiological relevance, these findings are not further discussed.

### Food intake

Chocolate snack intake after the scan session did not differ between the test sessions [mean (SEM): low load = 65.6 (5.9) g, high load = 68.1 (6.8) g; *t*(1,40) = –0.61; *P* = 0.546]. However, further analyses showed a significant interaction between attentional load and session order (low-distraction session first, high-distraction session first) for snack intake [*F*(1, 39) = 8.27, *P* = 0.007]. Specifically, food intake was significantly higher in the second, relative to the first, test session, independent of the attentional load of the test session [mean (SEM): session 1 = 61.2 (6.2) g, session 2 = 72.4 (6.4) g; *F*(1,40) = 8.68; *P* = 0.005; **Supplemental Figure 4**A; see Supplemental Figure 4B for a replication of this effect in a pilot study described in the Supplemental Methods: Behavioural pilot study]. It is likely that the interaction between load and session order for food intake is driven by a repetition effect. Given session order effects on food intake, session order was added as a between-subject factor to all other analyses (i.e., fMRI, glucose, and behavioral). None of the other reported results changed after correction for order, or interacted with order (all *P* > 0.1).

### Distraction-related brain–behavior correlations

**FIGURE 5:**
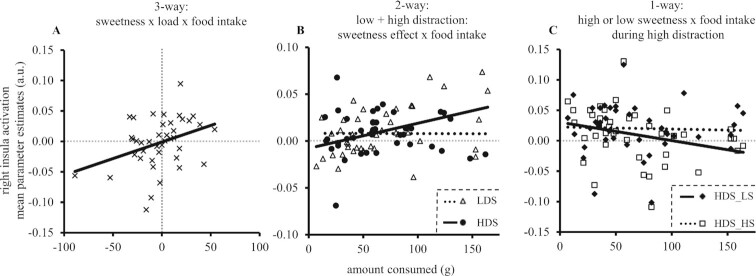
Brain–behavior correlations for the relation between taste-related (higher > lower sweetness) responses in the right insula, distraction (high, low attentional load), and ad libitum food intake (*n* = 41). (A) Significant brain–behaviour correlation at the highest level [3-way interaction between load (low–high distraction session), sweetness (higher–lower sweetness drink), and ad libitum intake (low–high distraction session); *r* = 0.36, *P* = 0.023]. (B) Separate correlations for the low (LDS; *r* = –0.01, *P* = 0.973) and high (HDS) distraction session (*r *= 0.45, *P* = 0.004; higher–lower sweetness). (C) Correlations in the high-distraction session only, for lower (LS; *r* = –0.30*, P* = 0.056*)* and higher (HS; *r* = –0.04, *P* = 0.823) sweetness separately. Less activation for the lower, but not the higher, sweetness drink in the high- but not low-distraction session seems to predict increased food intake in the high-distraction session. Mean parameter estimates are presented in arbitrary units (a.u.), ad libitum food intake in amount consumed in grams.

Interestingly, right insula activation during the fMRI task covaried with ad libitum intake of the chocolate snack 45 min after completing the task [load × sweetness × food intake: *F*(1,39) = 4.81, *r *= 0.36, *P* = 0.023; **Figure 5**A]. Subsequent analyses showed that this relation was present in the high- [sweetness × food intake: *F*(1,39) = 9.61, *r *= 0.45, *P* = 0.004] but not in the low-distraction session [sweetness × food intake: *F*(1,39) <1, *r* = –0.01, *P* = 0.973] (Figure 5B). More specifically, only activation for the lower-sweetness drink in the high-distraction session tended to predict how much participants would subsequently eat in the high-distraction session after fMRI [lower-sweetness drink, relation with food intake: *F*(1,39) = 3.87, *r* = –0.30, *P* = 0.056; higher-sweetness drink, relation with food intake: *F*(1,39) <1, *r* = –0.04, *P* = 0.823] (Figure 5C). Thus, in a continuous effect across the group, individuals in which high attentional load attenuated insula activation showed increased subsequent food intake and vice versa (Figure 5).

Including BMI and performance on the task as covariates did not change the above-reported pattern of findings. Finally, we did not find correlations for brain activation in the right insula and blood glucose concentrations, hunger, or fullness ratings, or any brain–behavior correlations for insula–OFC connectivity.

## Discussion

Distracted eating has been convincingly associated with increased food intake (6), but the underlying neurocognitive mechanisms remained elusive. Here, we examined how distraction—operationalized by varying attentional load—affects taste activation in, and connectivity between, primary and secondary taste-related brain areas (insula and OFC, respectively), blood glucose concentrations, subsequent chocolate snack intake, and appetite and thirst ratings.

As expected, higher compared with lower sweetness elicited differential BOLD responses in the insula: a small (2-voxel) cluster in the left insula and a larger cluster (12 voxels) in the right middle insula. We found no differential responses to sweetness in the OFC. These findings fit with recent work by Dalenberg et al. (14), who showed that the insular cortex processes the presence, pleasantness, and concentration of taste. More specifically, the right insular cortex dominated processing of taste concentration (intensity) signals, whereas the left insular cortex was more involved in representation of the presence of a taste stimulus and its pleasantness. This is in line with results from 2 other studies that also showed lateralization of intensity to the right insula (15, 16). Some studies have related activity of the right insula to processing of pleasantness; however, they did not control for effects of intensity (21, 39). In the present study, we varied taste intensity by comparing activation in response to a higher-sweetness versus a lower-sweetness drink and corrected for subjective liking differences of the 2 drinks at baseline. Crucially, this correction did not change the results of the higher > lower sweetness contrast. Therefore, the currently observed responses of the insula in the higher > lower sweetness comparison were mainly driven by differences in intensity, explaining the dominance of the right insula.

Importantly, we demonstrated that distraction attenuated taste-related functional connectivity between the right insula—found for the higher > lower sweetness contrast—and an area in the OFC. This OFC region is located in the caudomedial OFC (18), which is thought to be a relay between the anterior insula and the caudolateral OFC that is responsive to the pleasantness of taste (18). Another study manipulated pleasantness of chocolate milk and tomato juice through satiation and showed that pleasantness of the drinks correlated with taste activation in the left and right OFC, with the latter overlapping with the region found in our study (40). Thus, our findings suggest diminished functional coupling between primary and secondary taste cortices by distraction.

Our results further indicate that some individuals were more sensitive to distraction-related attenuation of taste-related activation in the right insula than others. Only when high attentional load affected taste processing of the lower-sweetness drink in the insula did subsequent food intake of participants increase. Previous work also showed large variation between participants in the effects of distraction on food intake (41, 42). Furthermore, the meta-analysis by Robinson et al. (6) showed that specifically highly disinhibited eaters are less likely to decrease their food intake after or during distraction. The interindividual variability found in our study is therefore not surprising and future studies should further investigate what drives these individual differences.

One study also investigated effects of cognitive load on food-related processing during fMRI [(13); note, however, that these authors manipulated working memory instead of attentional load]. In that study, higher cognitive load diminished nucleus accumbens responses during categorization of high- versus low-calorie food pictures. In addition, they showed that cognitive load altered the functional coupling between the nucleus accumbens and the right dorsolateral prefrontal cortex for high- versus low-calorie food pictures (13). However, these authors studied cognitive load effects on hedonic brain responses in the nucleus accumbens during categorization of high- and low-calorie food pictures versus object pictures as edible or inedible, in the absence of consumption during the task. By assessing actual taste processing during consumption of drinks in the scanner, we add to these previous findings that distraction attenuates connectivity in the taste network, and that the attenuating effect of attentional load on taste-related processing in the insula predicts subsequent food intake.

We found that responses for the lower-sweetness drink, in particular, predicted increases in later food intake under high load. Interestingly, a study by Hoffmann-Hensel et al. (43) found a similar effect when assessing the impact of cognitive load on fMRI responses to low- and high-calorie food odors with the same working memory load manipulation as by van der Wal and van Dillen (12). Their behavioral data revealed diminished perceived intensity for low- but not high-calorie food odors during high cognitive load. Similarly, higher cognitive load decreased OFC responses for low-calorie, but not the high-calorie, food odors. Their results could be explained by higher saliency of the high-calorie food odors and this may apply to our findings as well; as the higher-sweetness drink is more salient compared with the lower-sweetness drink due to its higher sweetness, attentional load might less easily suppress higher sweetness taste perception. It is, however, important to note that olfaction and gustation follow similar, but not identical, neural pathways [e.g., (43, 44)]. Nevertheless, the current and previous findings suggest that the saliency of odors and tastes plays a role in how distraction affects processing of food-related stimuli.

One limitation of our study is that the design was optimized for the primary outcome measure (i.e., the fMRI effects). Therefore, the distraction manipulation had to be relatively subtle (i.e., only varying the speed at which pictures were presented). Distractions such as watching a movie versus doing nothing in the MR scanner provide a less-controlled fMRI comparison with additional sources of variation (e.g., in perceptual, mood, and memory processes) than just varying attentional load. The relative subtleness of the distraction manipulation could explain why we did not find group effects of attentional load on food intake, or on appetite and thirst ratings.

In conclusion, by using fMRI during consumption, we found that distraction reduced functional connectivity between taste-processing areas and that distraction-related attenuation of taste-related processing in the insula predicted subsequent food intake. This provides a neurocognitive mechanism that improves our understanding of (the susceptibility for) overeating, and points to a potentially important role for undisrupted taste processing in overeating. A better understanding is essential for the successful prevention and treatment of overweight and obesity, where being mindful about the taste of food during consumption could perhaps be part of the solution. In our current—overly distracting—society, attentive eating might be more important than ever, to protect taste processing from being disrupted. Future studies should investigate the role of attention in neural taste processing in obesity.

## Supplementary Material

nqaa032_Supplemental_File

## Notes

This work was supported by a public–private Food, Cognition, and Behaviour grant funded by the Netherlands Organization for Scientific Research (NWO; grant 057-14-001) with financial support of Mars Wrigley. Mars Wrigley, which partly supported this work, did not influence the design or execution of the study, or interpretation of the results.

Supplemental Tables 1 and 2, Supplemental Methods, Supplemental Results, and Supplemental Figures 1–4 are available from the “Supplementary data” link in the online posting of the article and from the same link in the online table of contents at https://academic.oup.com/ajcn/.

Data described in the manuscript, code book, and analytic code will be made publicly and freely available without restriction at https://bit.ly/2nVZxVP.

Abbreviations used: AAL, Automated Anatomical Labeling; FoV, field of view; FWE, family-wise error; gPPI, generalized psychophysiological interaction; MNI, Montreal Neurological Institute; MR, magnetic resonance; OFC, orbitofrontal cortex; PPI, psychophysiological interaction; ROI, region-of-interest; TE, echo time; TR, repetition time; VAS, visual analog scale; WHR, waist-to-hip ratio.
