# Supplementary material for: Effects of distraction on taste-related neural processing: a cross-sectional fMRI study
Source: Am J Clin Nutr. 2020 Mar 16;111(5):950–61. doi: 10.1093/ajcn/nqaa032 (PMC7198299; doi:10.1093/ajcn/nqaa032)
Supplement: nqaa032_Supplemental_File [file nqaa032_supplemental_file.docx]

**Supplementary Methods**

*In- and exclusion criteria*

To be eligible for participation in the study, participants had to have a BMI within a range of 18.5 – 30.0, had to be within 18 – 35 years old, and right-handed. Exclusion criteria were current pregnancy; MRI-incompatibility; diabetes mellitus; history of hepatic, cardiac, respiratory, renal, cerebrovascular, endocrine, metabolic or pulmonary diseases; uncontrolled hypertension; neurological, psychiatric, or eating disorders; current strict dieting; restrained eating score ≥ 4.00 for females and ≥ 3.60 for males on the Dutch Eating Behaviour Questionnaire (DEBQ; (1), see **Supplementary Table 2**); current psychological or dietary treatment; taste or smell impairments; use of neuroleptica or other psychotropic medication; food allergies relevant to the study, deafness, blindness, and sensori-motor handicaps; drug, alcohol or nicotine addiction; inadequate command of both Dutch and English, and a change in body weight of more than 5 kg in the past two months.

*Gustatory pilot study*

To avoid differences in liking between the lower and higher sweetness chocolate drinks participants received during the task in the MR-scanner, we performed a pilot study in which 7 solutions of cocoa powder (Blooker, 2 g), dextrine-maltose (Fantomalt, 9 g) in whole milk (3.5% fat/100 g), and liquid non-caloric sweetener (Natrena, ranging from 0.0867 to 1.955 g, in steps of 0.2669 g) were rated by 10 participants (who did not participate in the current study) on a 100mm VAS, labelled “not at all liked” and “very much liked”. Using the average liking rating from this pilot study, the concentrations for lower and higher sweetness drinks were determined such that both the lower and higher intensity drink were at equal distance from the optimum. The two concentrations chosen for the lower and higher sweetness chocolate milk contained 0.0867 and 1.5457 g of Natrena per 100 g whole milk.

A similar approach was used to determine the neutral solution. Based on previous work by van Veldhuizen et al. (2), we created four solutions containing 2.5 mM sodium bicarbonate and 25 mM potassium chloride in water. We also created three weaker versions at 25%, 50% and 75% of the original concentration. The most neutral concentration (closest to a liking rating of 5 on a scale of 0 to 10) was used, which was the solution at 50% of the original concentration.

*D-prime*

To calculate participants’ task performance, we used the sensitivity index d-prime (*d’*). From the task, four types of response were obtained: hits (target was detected correctly), miss (target was present, but the participant incorrectly indicated there was no target), false alarms (participant indicated a target was present when there was not), and correct rejections (participant correctly indicated there was no target). D-prime was calculated using the formula: *d’* = Z_Hit_ – Z_FA_ (3,4), where “Hit” represents the proportion of hits when a target was present (hits/(hits + misses)), also known as the hit rate, and “FA” represents the proportion of false alarms when a target was absent (false alarms/(false alarms + correct rejections)), the false-alarm rate. D-prime is then calculated by taking the difference between the Z-transforms of these two rates. The Z-transformation was done using the statistical formula NORMSINV(Hit)– NORMSINV(FA) in Matlab (2016a). To avoid *d’* scores reaching -∞ or +∞, perfect scores were adjusted by subtracting 0.0025 from the hit rate, and adding 0.0025 to the false alarm rate. This correction resulted in maximum *d’*-scores of +5.61 (100% hits, 0% false alarms), and minimum scores of

-5.61 (0% hits, 100% FA).

*Video analysis of swallow movements*

Recordings of participants’ swallow movements were made with an in-bore camera and infrared LED light (MRC Systems GMbH). Matlab software (Matlab, version 2016a) was used to detect the circle-shaped marker placed on the participant’s neck in the video. After detection, a rectangular frame-of-interest was determined around the marker using the subject-specific centre coordinates and radius of the marker to reduce the search area. To determine the size of the frame of interest, the x- and y-coordinates of the marker’s centre and its radius were multiplied by two. Next, the marker’s radius was added to or subtracted from the centre coordinates to calculate the boundaries of the frame of interest in the –x, +x, -y and +y direction. To time lock the video data to the MR scanner pulses, a beep was recorded at the onset of the first scanner pulse. Subsequently, frame-to-frame video intensity differences were extracted from the videos and coupled with the onsets of trials to determine when participants swallowed the chocolate milk. To detect on- an offsets of the swallows, the Hilbert transform was used, a function that can determine the envelope of a waveform in an analytical signal ([Matlab](https://nl.mathworks.com/help/signal/ug/envelope-extraction-using-the-analytic-signal.html) version 2016a).

*Liking and ideal sweetness ratings*

To test for pre-experimental differences in liking of the three drinks we performed a repeated-measures ANOVA with within-subject factor Drink Type (lower sweetness, higher sweetness, neutral) on the mean baseline ratings. Furthermore, we performed a repeated-measures ANOVA with within-subject factors Load (low, high), Drink Type (lower sweetness, higher sweetness, neutral), and Time (t_0(1)_, t_0(2)_, t_30(1)_, t_30(2)_) to test whether liking decreased significantly over time for the drinks. Moreover, we exploratively assessed whether liking ratings were affected by attentional load.

With respect to the ratings on how well the lower and higher sweetness drinks matched participants’ ideal sweetness, we aimed to show that both the lower and higher sweetness drinks were at equal distance from the optimum. To test this, we calculated the absolute difference from the optimum by subtracting the optimum (a rating of 5) from the lower and higher sweetness ratings. Subsequently, a paired samples t-test was used to test whether these mean ratings were significantly different. Exploratory, we assessed whether there were changes in these ratings over time, or as a function of attentional load.

*Desire for something sweet or savoury*

Before and after the task in the MR scanner, we asked participants how much they desired “something sweet” and “something savoury”. If sensory specific satiety was successfully induced, we expected their desire for something sweet, but not savoury, to decrease significantly during the task. To test this, we executed a repeated measures ANOVA with within-subject factors Load (low, high), Taste (sweet, savoury) and Time (t_0_, t_30_) and assessed the interaction effect between Taste and Time to test whether participants’ satiety decreased specifically for the sweet taste.

*Behavioural pilot study*

Prior to the current study, we performed a behavioural pilot study with a similar set-up as the current study in 31 participants, to optimize the study’s design. They performed the same visual detection task on two separate test sessions (high, low distraction) in an MRI-like set-up in a behavioural lab. However, no actual fMRI scanning was performed. To mimic the MRI-set-up, participants lay on a table and heard MRI-sounds through headphones during the experiment. Participants performed 80 trials on the visual detection task during computer-paced consumption of chocolate milk; however, there were no trials during which the neutral solution was administered via the gustometer. Participants performed 40 trials in the higher and lower sweetness condition, instead of 32 in the current study. As a result, participants consumed 150 g of each chocolate milk during the task, instead of 120 g. Eighty (instead of 90) percent of trials were of high frequency load, and 20% (instead of 10%) of low frequency load. Instead of a chocolate snack, subjects consumed chocolate milk *ad libitum*. No blood glucose measurements were taken. In line with the results of the current study, we found that participants consumed more chocolate milk on the second test session than on the first (M_session1_ = 73.2(10.3) g, M_session2_ = 111.8(19.0) g, *F*(1,29) = 8.24, *p* = 0.008, **Supplementary Figure 2B**).

**Supplementary Results**

*Liking and ideal sweetness ratings*

We expected liking ratings to decrease significantly for the lower and higher sweetness, but not the neutral, drink over the time course of the task in the MR scanner as a result of sensory-specific satiety. Results show a main effect of Time (*F*(1,19) = 6.49, *p* = 0.003)), indeed reflecting significant decreases in liking after the task compared to baseline for the lower and higher sweetness drinks, but not for the neutral drink (see Supplementary Table 1 for means and standard errors / deviations, and statistics). Explorative analysis of the effect of distraction (attentional load) on liking ratings showed no significant results (interaction effect of Load, Drink Type and Time: *F*(1,16) <1, *p* = 0.795). At baseline, participants liked the higher sweetness drink significantly more than neutral drink (high sweet drink, M = 6.1(0.3); neutral drink, M = 4.5(0.5), *t*(1,35) = 2.64, *p* = 0.012). There was no difference in liking ratings between the lower sweetness and neutral drink (low sweet drink, M = 5.4(0.3); neutral drink, M = 4.5(0.5), *t*(1,34) = 1.73, *p* = 0.092).

Furthermore, we assessed whether participants rated both the lower and higher sweetness drink equally far from the optimum (a rating of 5) in terms of how well the drinks matched their ideal sweetness. As expected, we found no significant differences at baseline on this measure for the lower relative to the higher sweetness drink, showing that the lower sweetness drink was perceived equally far from participants’ ideal sweetness as the higher sweetness drink (mean difference from optimum: higher sweetness drink, M = 1.5(0.2); lower sweetness drink, M = 1.0(0.2), *t*(1,26) = 1.47, *p* = 0.153). There were no significant decreases on these ratings over time *F*(1,14) = 1.42, *p* = 0.280, Supplementary Table 1), nor as a function of distraction *F*(1,14) <1, *p* = 0.430).

*Desire for something sweet or savoury*

A repeated measures ANOVA with within-subject factors Load (low, high), Taste (sweet, savoury) and Time (t_0_, t_30_) revealed significant main effects of Taste, Time, and an interaction effect of Taste and Time on participants’ desire for something sweet or savoury (Supplementary Table 1). The main effect of Taste reflects a larger overall desire for “something savoury” relative to “something sweet” (*F*(1,37) = 92.45, *p* < 0.001), and the main effect of Time indicates a significant overall reduction in desire (*F*(1,37) = 23.51, *p* < 0.001). Finally, the significant Taste x Time interaction reflected a larger decrease in desire for the sweet taste over time relative to the savoury taste, showing successful induction of sensory specific satiety for the sweet taste (*F*(1,37) = 17.16, *p* < 0.001).

*Blood glucose concentrations*

Distraction (attentional load) did not significantly affect glucose increases over time (*t*(1, 260.19) = 1.81, p = 0.072). However, an exploratory analysis did reveal a significant distraction-related decreased rise in glucose concentrations at t = 75 relative to baseline (low distraction session: M_t75-t0_ = 2.94(1.48) mmol/L, high distraction session: M_t75-t0_ = 2.50(1.32) mmol/L, *t*(1, 113.96) = 2.09, p = 0.039). Nevertheless, *post-hoc* simple effects (paired samples t-test for low versus high load at time points t0, t30, t50, and t75) showed the effect was not significant at separate time points (t0, t30, t50: all p>.1; t75: *p* = 0.087)*.* The distraction-induced attenuation of blood glucose rise correlated negatively with changes in hunger ratings (*t*(1, 37) = -3.44, coefficient of correlation, sample: *r* = -0.50*,* p = 0.002). This correlation was driven by a significant effect on the low distraction session, on which increases in blood glucose rise were related to decreased hunger ratings (*t*(1, 37) = -2.14, *r* = -0.34*,* p = 0.039). On the high distraction session, there was no correlation between hunger and glucose (*t*(1, 37) = 0.56, *r* = 0.09*,* p *=* 0.581). The correlations between glucose and hunger were not significant when taking all four time points into account (all *p*>0.5).

**Supplementary Tables**

**Supplementary Table 1.** Neuropsychological measurements

|  | Mean | Standard deviation | Minimum | Maximum |
| --- | --- | --- | --- | --- |
| BIS | 15.5 | 2.9 | 10.0 | 24.0 |
| BAS | 24.0 | 4.9 | 18.0 | 35.0 |
| BIS-11 | 68.9 | 5.2 | 59.0 | 80.0 |
| Kirby | 0.006 | 0.008 | 0.0002 | 0.03 |
| BES | 24.2 | 5.1 | 16.0 | 35.0 |
| FFQ-DHD | 53.4 | 12.4 | 25.0 | 79.0 |
| DEBQ |  |  |  |  |
| *Restraint* | 2.3 | 0.7 | 1.0 | 3.9 |
| *Emotional* | 2.3 | 0.6 | 1.2 | 3.5 |
| *External* | 3.3 | 0.5 | 2.5 | 4.3 |
| PFS | 36.2 | 9.9 | 21.0 | 60.0 |

*BIS/BAS:* Behavioural Inhibition System/Behavioral Approach System questionnaire; *BES*: Binge Eating Scale; *BIS-11*: Baratt Impulsiveness Scale-11; *DEBQ*: Dutch Eating Behaviour Questionnaire; *FFQ-DHD*: Food Frequency Questionnaire, Dutch Healthy Diet; *Kirby*: delayed reward discounting questionnaire; *PFS:* Power of Food Scale. N=41.

**Supplementary Table 2.** Self-reported liking, ideal sweetness, and desire for something sweet or savoury ratings, averaged over Load (high, low). Means and standard errors per time point, and Time statistics.

|  |  | t _(0)_ | t _(5)_ | t _(10)_ | t _(30)_ | p | F |
| --- | --- | --- | --- | --- | --- | --- | --- |
| *Liking ratings* | | | | | | | |
| Low sweet drink |  | 5.4(0.3) | 4.6(0.3) | 3.6(0.3) | 3.9(0.3) | <0.001 | 13.92 |
| High sweet drink |  | 6.1(0.3) | 5.9(0.3) | 5.2(0.4) | 5.1(0.4) | 0.025 | 3.58 |
| Neutral solution |  | 4.5(0.5) | 4.6(0.5) | 4.7(0.5) | 4.8(0.5) | 0.639 | <1 |
| *Ideal sweetness ratings* | | | | | | | |
| Low sweet drink |  | 3.9(0.2) | 3.8(0.3) | 3.5(0.3) | 3.7(0.3) | 0.364 | 1.37 |
| High sweet drink |  | 6.4(0.3) | 6.6(0.3) | 6.6(0.3) | 6.6(0.3) | 0.878 | <1 |
| *Desire for something sweet or savoury* | | | | | | | |
| Sweet |  | 5.6(0.3) | - | - | 3.7(0.3) | <0.001 | 36.26 |
| Savoury |  | 7.2(0.2) | - | - | 7.0(0.3) | ns. | <1 |

N = 41

**Supplementary Figures**

**Supplementary Figure 1.** Flow Diagram

Excluded after intake session (n=6)

♦ Refused to participate (n=5)

♦ Not MRI compatible (n=1)

Lost to follow-up

♦ Not MRI compatible at session 2 (n=1)

Last day of data collection: 22-9-2017

Analyzed (n=41)
♦ Excluded from analysis due to technical problems (n=4)

Total recruited (n=46)

Assessed for eligibility (n=52)

Recruitment 14-7-2016 till 24-7-2017


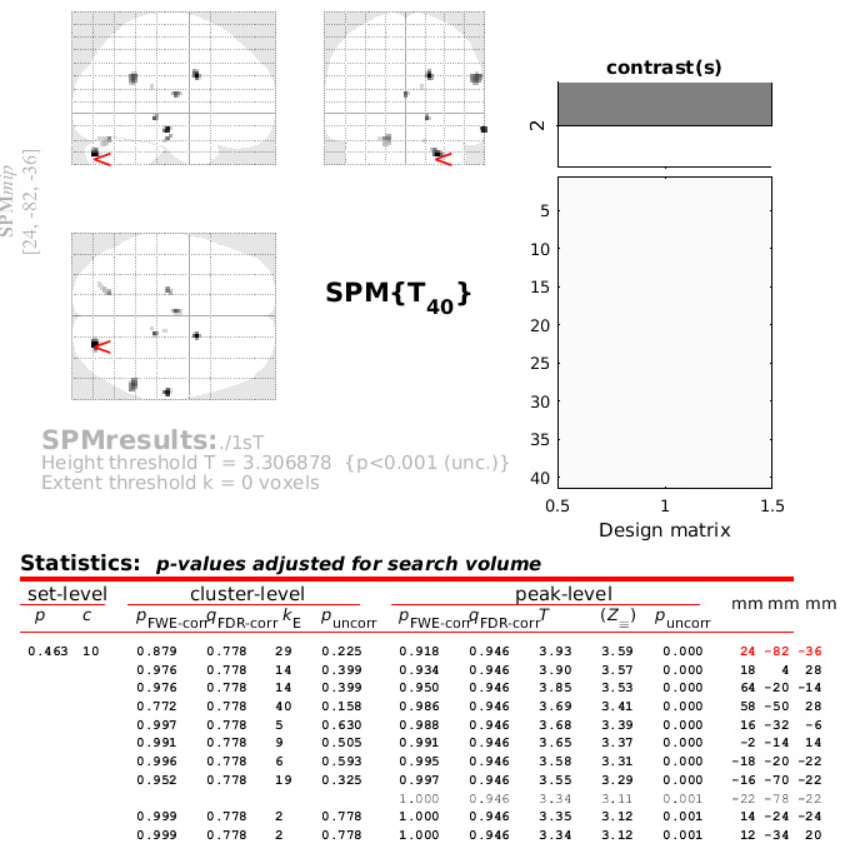


**Supplementary Figure 2.** Results of the exploratory analysis on the effect of distraction (low>high load) on sweetness processing (high>low sweetness) at the whole-brain level (*p*<0.001, uncorrected).

**
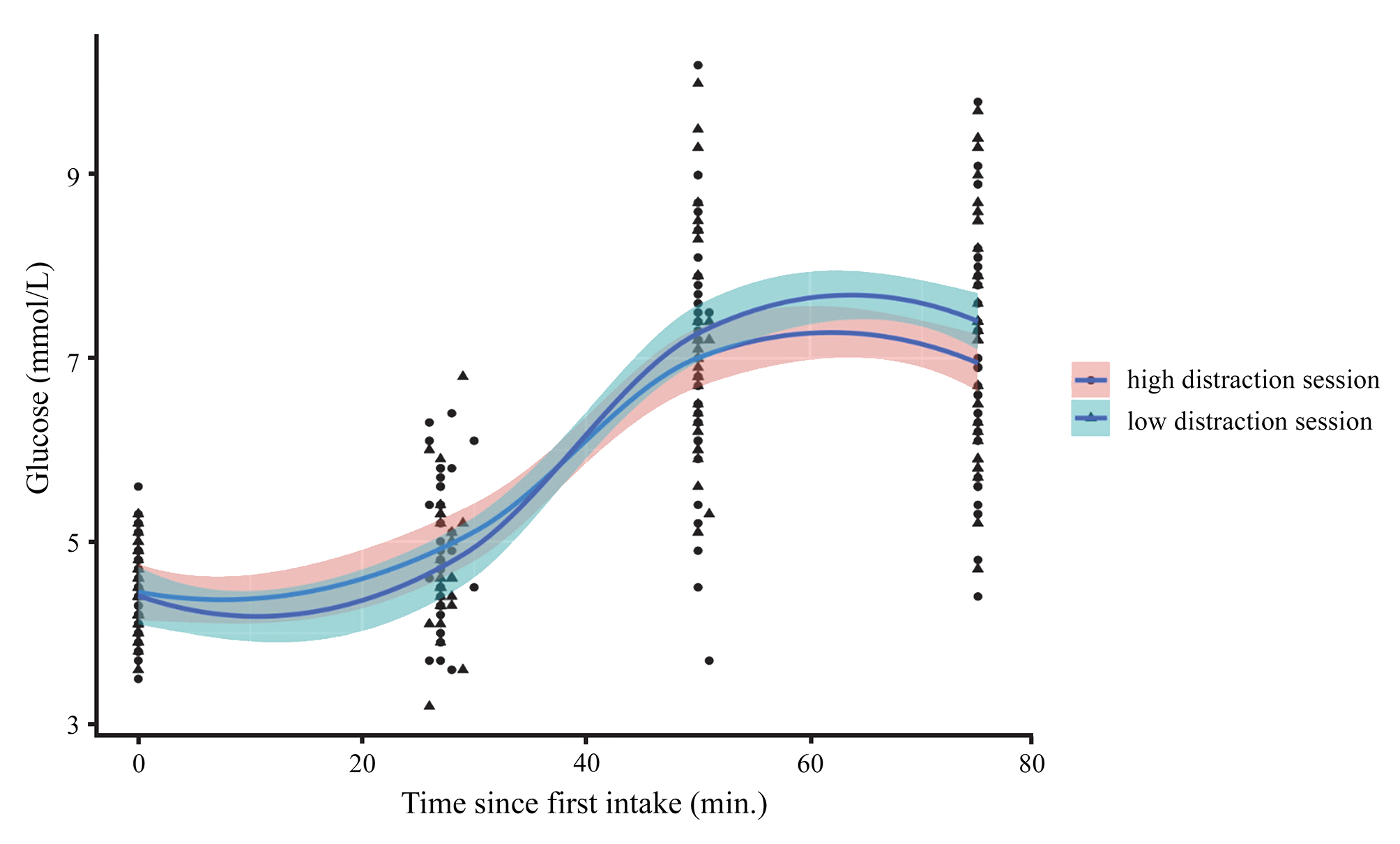
**

**Supplementary Figure 3.** Blood glucose response (means, standard errors and individual data points) to the chocolate milk per session (high, low distraction session) for each time point (at baseline (0 g of chocolate milk consumed), right after the task (240 g consumed), 50 and 75 minutes after baseline) in mmol/L. Distraction (attentional load) did not significantly affect glucose increases over time (p = 0.072). N = 41. Loess lines of best fit were used to fit the data.

**
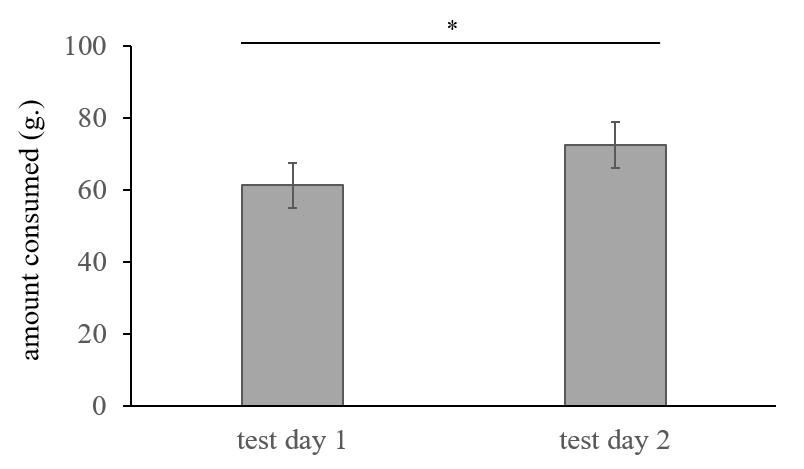
**
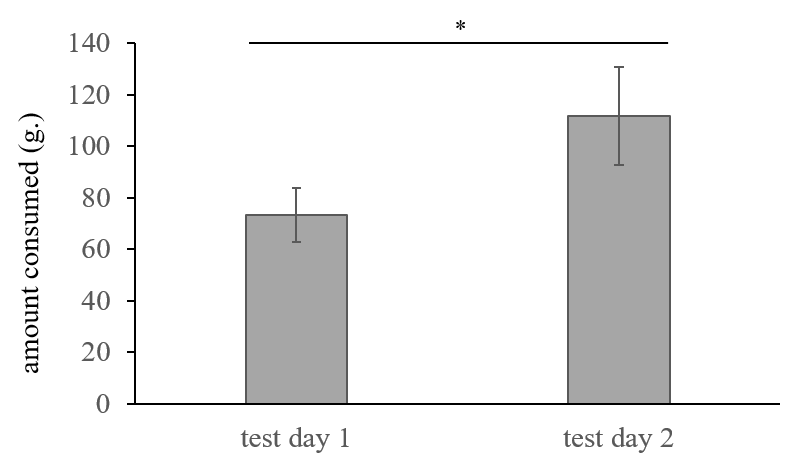


B

A

**Supplementary Figure 4.** *Ad libitum* intake of the chocolate snack (current study) or milk (pilot study) for each test day (test day 1, test day 2), independent of Distraction (attentional load).Error bars depict standard error of the mean. Panel A) Intake in the current study. The mean amount consumed (g) was significantly higher on the second test day. The asterisk indicates *p* = 0.008. Panel B) Intake of the chocolate milk in the pilot study. In line with the results of the current study, the mean amount consumed (g) was significantly higher on the second test day. The asterisk indicates *p* = 0.005.

**References for On-line Supplementary Material**

1. Van Strien T, Frijters J, Bergers G, Defares PB. The Dutch Eating Behavior Questionnaire (DEBQ) for assessment of restrained, emotional, and external eating behavior. Int J Eat Disord [Internet]. 1986;5:295–315. Available from: http://onlinelibrary.wiley.com/doi/10.1002/1098-108X(198602)5:2%3C295::AID-EAT2260050209%3E3.0.CO;2-T/full

2. Veldhuizen MG, Nachtigal D, Teulings L, Gitelman DR, Small DM. The insular taste cortex contributes to odor quality coding. Front Hum Neurosci. 2010;4:1–11.

3. Haatveit BC, Sundet K, Hugdahl K, Ueland T, Andreassen OA, Haatveit BC, Sundet K, Hugdahl K, Ueland T, Haatveit BC, et al. The validity of d prime as a working memory index : Results from the “ Bergen n -back task ” The validity of d prime as a working memory index : Results from the “ Bergen n -back task .” 2010;3395.

4. Snodgrass JG, Macmillan A. Response Bias : Characteristics of Detection Theory , Threshold Theory , and " Nonparametric " Indexes. 1990;107:401–13.
